# The weaponization of professionalism against physicians of color

**DOI:** 10.1186/s12960-024-00931-y

**Published:** 2024-07-16

**Authors:** Maria Borrero, Lauren Kiel, Inas Abuali, Zalaya K. Ivy, Narjust Florez

**Affiliations:** 1 Division of Hematology/Oncology, University of Minnesota, 420 Delaware Street SE, Mayo Mail Code 480, Minneapolis, MN 55455 United States of America; 2 Dana-Farber Cancer Institute, Boston, MA United States of America; 3 Division of Hematology/Oncology, Massachusetts General Hospital, Boston, MA United States of America; 4 Division of Hematology/Oncology, Department of Medicine, Dana-Farber Cancer Institute, Harvard Medical School, Boston, MA United States of America

**Keywords:** Cultural diversity, Professionalism, Racism, Sexism

## Abstract

Though we have made ample advances in the field of medicine in recent years, our idea of professionalism continues to be based on the standard of how white men dressed in the nineteenth century. Such a standard of professionalism not only perpetuates gender bias, but also aims to remove the culture, traditions, and behaviors of minority groups with the goal of molding these individuals to resemble the majority, preventing ‘Afro’ heritage from entering medicine. By contextualizing our own experiences in the medical setting as physicians of color in the context of a variety of supporting literature, we provide an overview of professionalism, its role in medicine, the double standard faced by women, and how it continues to be weaponized against physicians of racial, ethnic, and religious minorities. We advocate for minority physicians to embrace their authenticity and for institutions to develop policies that openly, firmly, and enthusiastically welcome physicians of all ethnicities, religions, and genders. Positionality Statement: In the editorial you are about to read, we, the authors, collectively bring a rich tapestry of backgrounds and experiences to our discussion on healthcare disparities. Our team consists of two Hispanic/Latina oncologists, one Middle Eastern oncologist, one Black/Caribbean-American hematologist, and one White pre-medical student with Middle Eastern heritage. Our diverse backgrounds inform our perspectives and enhance our understanding of the complex and multifaceted nature of healthcare. We are united by a shared commitment to justice, equity, and the belief that every patient deserves high-quality care, regardless of their background. This editorial is informed by our professional expertise, personal experiences, and the diverse communities we serve, aiming to highlight the critical need for inclusivity and representation in healthcare. By acknowledging our positionality, we hope to provide a comprehensive and empathetic analysis that not only identifies the challenges but also offers actionable solutions to improve healthcare outcomes for all. We recognize the power of diversity in fostering innovation and driving positive change, and we are dedicated to using our voices and positions to advocate for a more equitable healthcare system.

## What defines professionalism?

Professionalism is defined as the conduct, aims, or qualities that characterize a profession or a professional person. As it pertains to medicine, in 1999, the ACGME included professionalism as one of six general competencies that we must achieve during medical training. That same year, the American Board of Internal Medicine Foundation, American College of Physicians, and European Federation of Internal Medicine created the Charter of Medical Professionalism, now adopted by over 100 physician organizations, to define three fundamental principles of professionalism: patient welfare, respect for patient autonomy, and social justice. Interestingly, the charter does not mention a set of rules regarding our outward appearance as physicians, religious practices, social behaviors, and interpersonal skills. However, the way we look, how we dress, how we style our hair, and our outfit’s colors, all play a significant role in medicine’s unspoken definition of professionalism. As a medical society shaped by marginalization, unconscious and conscious biases, and guided by subjective opinions of superiors in medical training, we have set our own definition of professionalism, one that is often weaponized against us physicians of color. But if our traditional cultural clothing, natural hair, religious attire, and acceptance of our authentic selves do not align with the current “standard of care” of professional appearance, does that mean we pose a threat to medicine?

## The colors of professionalism

The first time I was invited to a medical conference in the summer of 2009 in Austin, TX, I went shopping for attire with my sister and mother, two Latinas whose wardrobes are always overflowing with bright colors, bold necklaces, and the newest fashion trend. I proudly packed a suitcase full of outfits we picked, only to arrive at the conference and realize no one was dressed like me. I was furious at my sister for making me look “so Latina” and was determined to fit in moving forward, wearing attire that wouldn’t make me stand out as a woman of color: black, gray, and navy colored clothing only. But why did these colors become the status quo?

Outwardly required or implied, attire is one way to represent our professional identity. A quick Internet search will suggest wearing black, gray, white, and navy blues to professional interviews to display credibility, strength, authority, and organization, and avoid brighter shades such as oranges, reds, and multi-colored patterns, which may be perceived as jarring and unprofessional. In fact, formal attire is associated with perceptions of physician trust and confidence [1], while less formal attire may convey a lack of competence or insufficient training. Unsurprisingly, a recent literature review of 30 investigations found that physicians wearing white coats and formal attire, such as collared shirts, straight or tucked back hair, ties, slacks, skirts, or suit pants were perceived by patients as more ‘trustworthy’, regardless of their level of training or experience [1]. A 2022 investigation with over 9000 participants also found strong preference for the white coat and solid, dark, formal attire, especially in the United States [2] (with brighter colored surgical scrubs as an exception [1]), while other research found less preference for “feminine” wear such as prominent ruffles, dangling earrings, and patterned hose [3]. Though patients may in fact prefer traditional “professional” attire, we have to ask ourselves: are these responses not based on perpetuated stereotypes? After all, they arise from old practices of removing culture, traditions, and behaviors from minority groups with the goal of molding us to resemble the majority; preventing ‘Afro’ heritage from entering medicine. Though more research is needed, we posit that for cultures that celebrate their heritage through ornate patterns, bright colors, unique designs, or additional garments, “remaining professional” may seem instead like subduing your identity.

## The double standard of the dress code

The origins of professional attire can be traced back to the nineteenth century in Europe and North America, where the business suit became widely adopted as the standard professional attire for men. Despite the passage of time, the addition of women into our workforce, and the continued diversification of our population, we have continued to perpetuate a professionalism standard for attire based on how white men dressed in the nineteenth century. In fact, in a study [4] exploring the public perception of physician attire, men wearing business wear or simply hospital scrubs were perceived as significantly more professional than women wearing the same outfit. Unsurprisingly, the male model was also more likely to be identified as a physician than the female, regardless of attire, including the presence of a white coat. Compared to male hematology and oncology fellows, female fellows reported experiencing more gender bias and using techniques such as wearing a white coat, emphasizing a “professional” look, and ensuring that “Doctor” was clearly written on their badge, to combat the daily gender bias and challenges experienced in the work environment [5]. Thus, as we women continue to be subject to the rampant gender bias present in medicine even when adhering to societal expectations of professionalism, we ask our male colleagues: the next time that patients continue to look at you and not acknowledge us due to your identified gender, speak up, redirect. Be our ally.

## Hairstyles used against women of color

For us as Black women, hairstyle choice is a core component of “appearance labor”, as we are inherently in the position to contend with the reality of a hair penalty due to how choosing to adorn Afrocentric hairstyles is deemed “less professional and more dominant” than choosing Eurocentric hairstyles [6]. Recently, the issue of discrimination against our hair garnered federal attention when the Creating a Respectful and Open World for Natural Hair Act (“CROWN Act”) was passed in the House of Representatives but subsequently blocked by the U.S. Senate in December 2022. Though this Act later became law in 23 US states, other states continue to seek its enactment. Encompassed in this hair dilemma are contending notions of oppression associated with discrimination based on hair texture/hairstyles versus the utilization of hair to cultivate a standard of beauty that values our Black hair. Both explicit and implicit messages to alter hairstyle choices perpetuates an anxiety in us that our natural hair is not acceptable or professional enough, for as Black women, hair is an extension of our identity. Just as it is unreasonable for someone with blue eyes to change their eye color, the expectation that we as Black women must change our hair to conform to Eurocentric beauty standards is dehumanizing. We invite our colleagues to be mindful of their own unconscious bias against our natural hair. Next time you point out that our hair looks best in a certain style, ask yourself: are you referring to the style that most closely mirrors Eurocentric beauty standards?

## Religious attire in the workplace

Dress code policies in healthcare should rely on evidence-based practices that promote patient safety, rather than archaic “professionalism” notions based on Eurocentric ideals. Occasionally, patient preferences are cited to justify discriminatory dress code policies, with restrictions only on certain ethnic and religious minorities [7].

Banning religious headwear in operating rooms while allowing homemade cloth scrub caps [8] is unfortunately prevalent, despite lack of scientific evidence to support increased infection risk. Furthermore, we hijab-wearing staff are not provided with educational resources on how to scrub in the operating room and how to wear our personal protective equipment (PPE) in an inclusive manner to ensure comfort while maintaining appropriate infection control practices. Limiting surgical masks to those with ear loops only is another example of how we as hijab-wearing staff are excluded and expected to make personal adjustments to our religious attire to conform to available PPE.

Muslim physicians deciding not to wear a hijab in the workplace cited reasons such as patients’ and colleagues’ micro- and macroaggressions regarding their attire, in addition to prejudices impacting hiring and promotion [9]. This marginalization also extends to other religious minorities with visible religious articles of clothing such as Sikh men wearing turbans and Jewish men wearing Kippahs. It is imperative that our institutional dress code policies strive to embody cultural humility and embrace an ethnically and religiously diverse society.

## Diversity without inclusion equals trauma

Medical institutions need to create environments and policies that openly, firmly, and enthusiastically welcome physicians of all ethnicities, religions, and genders. We cannot pretend to be diverse while forcing majority group behaviors and practices, including our outward appearance, onto our minority trainees and faculty. We must redefine policies, including dress codes, and be intentional about our unconscious biases to create inclusive environments for our present and future workforces.

## Conclusions

For far too long, we as minority physicians have conditioned ourselves into thinking that somehow being our authentic selves is not professional and will negatively impact our workforce interactions or even patient care. Nevertheless, our authenticity is one of our greatest assets; it gives us the ability to develop higher trust and satisfaction with our ever-growing diverse patient population, for they see themselves in us [10]. It is time we embrace this narrative. To those that don’t fit the status quo, you belong here. Your floral print, your bright colors, your curly/coily hair, your hijab, your hoop earrings—they all belong.

## Data Availability

Not applicable.

## Abbreviations

ACGME: Accreditation Council for Graduate Medical Education
PPE: Personal protective equipment
